# Exploring Solitary Fibrous Tumors at a Tertiary Cancer Center: Clinicopathological and Immunomorphologic Profile

**DOI:** 10.7759/cureus.56899

**Published:** 2024-03-25

**Authors:** Zachariah Chowdhury, Soumya Mishrikotkar, Pritika Nehra, Shashikant Patne, Mayank Tripathi

**Affiliations:** 1 Oncopathology, Mahamana Pandit Madan Mohan Malviya Cancer Centre (MPMMCC) and Homi Bhabha Cancer Hospital (HBCH) (Tata Memorial Hospital), Varanasi, IND; 2 Surgical Oncology, Mahamana Pandit Madan Mohan Malviya Cancer Centre (MPMMCC) and Homi Bhabha Cancer Hospital (HBCH) (Tata Memorial Hospital), Varanasi, IND

**Keywords:** p53, bcor, risk assessment, salas, demicco, stat6, solitary fibrous tumors

## Abstract

Background

Solitary fibrous tumor (SFT) is a distinct fibroblastic tumor that can occur at any anatomical site and can manifest a variety of histopathological features. NAB2-STAT6 gene fusion has recently emerged as a sensitive and specific molecular marker and its surrogate on immunohistochemistry, STAT6 has also displayed considerable efficacy. Nevertheless, its histologic diversity can result in diagnostic challenges, especially when classic features are not apparent.

Methods

A retrospective study was conducted at a tertiary cancer centre in North India over 3 years to document the clinicopathologic and immunomorphologic profile of SFTs. Immunohistochemical analysis of BCOR and p53 were gauged additionally and patients were stratified according to Modified Demicco and Salas criteria for risk of metastasis.

Results

Sixteen patients of SFT were identified, affecting middle-aged men and women equally. Though lung/pleura are known to be involved commonly, SFT affects other sites such as the kidney, brain, buccal mucosa, liver, and penis as well. The majority endured localized disease while a lesser number suffered locoregional/distant spread. Two patients revealed features of a malignant profile. Risk stratification according to the Modified Demicco and Salas criteria evinced comparable results. No discernible relationship however was highlighted between the immunohistochemical expression of BCOR, p53, and any significant SFT parameter.

Conclusion

Although SFTs are very rare substantially benign mesenchymal neoplasms, pathologists must be conversant with their histological diversity and be vigilant of their malignant attributes. The worth of STAT6 immunohistochemistry for precise diagnosis and long-term studies for delineating clinical behavior cannot be overemphasized.

## Introduction

Solitary fibrous tumor (SFT) is a rare soft tissue tumor of mesenchymal origin. First identified in the pleura in 1931 by Klemperer and Coleman, these tumors were coined hemangiopericytoma by Stout and Murray [1]. Having been known by a variety of names in the past, including benign mesothelioma, localized mesothelioma, solitary fibrous mesothelioma, and localized fibrous tumor, to mention a few, hemangiopericytoma has since been replaced with the term solitary fibrous tumor as a result of the identification of the SFT fusion gene [2]. They have posed special diagnostic and therapeutic difficulties since they share many histological and gross characteristics with other soft tissue tumors.

SFTs are categorized as intermediate biological potential with a low risk of metastasis and relatively indolent course under the 2020 WHO Classification. While the vast majority of SFTs are benign, around 5-45% exhibit aggressive clinical behavior, which can result in local recurrence and/or metastatic disease [3]. Criteria for malignancy have not been consistent between published series. The majority of data, which has been acquired from small retrospective series and case reports due to the low incidence of SFT has made it challenging to create specialized diagnostic approaches and treatment planning. The 2020 WHO classification favored the use of risk stratification models such as those prescribed by Demicco and Sala over traditional terms like “typical” or “malignant” to assess prognosis in SFT, considering them to be more effective tools to assess the behavior of these tumors [4]. Studies assessing the risk factors published in the English literature have been few and far between. The authors embarked on this study to chronicle the clinicopathologic features of SFT, illustrate the associated diagnostic pitfalls and utilitarian pearls, and endeavor risk stratification of these patients. Furthermore, the role of p53 and BCOR expression in these cases was investigated with the use of immunohistochemistry.

## Materials and methods

This was a retrospective study, conducted after approval from the Institutional Ethics Committee (IEC) vide letter no OIEC/11000661/2023/00002. Data was collected for a period of four years (2019-2023). The Institutional Electronic Search Engine was used for retrieving cases using the keywords “solitary fibrous tumor”. Informed telephonic consent was obtained from all the individual participants included in the study. Clinical details of the patients including age, sex, symptoms, radiology, treatment, and follow up were obtained from the electronic medical records. All the cases were screened and selected as per histopathologic and immunohistochemical features, including positive immunostaining for STAT6 and CD34. The cases were stratified according to the modified Demicco risk stratification system and the Salas criteria for risk of metastasis (Salas (MET)), as shown in Table 1 [5].

**Table 1:** Modified Demicco risk stratification system and the Salas criteria for risk of metastasis (Salas (MET)) Reference: Demicco et al. [5]

Modified Demicco risk stratification system	
Risk factor	Score
1. Age	
< 55	0
≥ 55	1
2. Tumor size (cm)	
< 5	0
5 to < 10	1
10-15	2
≥ 15	3
3. Mitotic count (/10 high-power fields)	
0	0
1-3	1
≥ 4	2
4. Tumor necrosis	
< 10%	0
≥ 10%	1
Risk class	Total score
Low	0-3
Intermediate	4-5
High	6-7
Salas criteria for risk of metastasis [5]	
Risk factor	Score
1. Mitosis	
≤ 4/10 high-power fields (HPF)	0
> 4/10 HPF	1
2. Age	
< 60 years	0
≥ 60 years	1
3. Location	
Limbs (distal extremities)	1
Others	0
Risk class	Total score
Very low	0
Low	1
Intermediate	2
High	3

Immunohistochemistry (IHC) staining was performed by polymer detection technique on 3 μm thick sections mounted on tissue-bound coated slides. STAT6 rabbit polyclonal antibody, directed against the C terminus of STAT6 (EP325; Santa Cruz Biotechnology, Santa Cruz, California, USA), with dilution 1:50, was used (Table 2).

**Table 2:** Immunohistochemistry antibodies used in our study

	Antibody	Clone	Dilution	Source
	STAT6	EP325	1:50	Cell Marque (Sigma Aldrich)
	CD34	EP88	1:200	Cell Marque
	CD99	EP8	1:50	Vitros
	BCOR	C10	1:50	Zeta
	p53	SP5	1:100	Cell Marque
	S100	4C4.9	1:500	Cell Marque
	SMA	1A4	1:200	Cell Marque
	Ki67	30-9	Ready to use	Roche (Ventana)
	Desmin	D33	1:100	DAKO
	CD31	JC70A	1:100	DAKO
	HMB45	HMB45	1:100	DAKO
	h-caldesmon	h-HCD	1:50	Cell Marque
	TFE3	MRQ37	Ready to use	Cell Marque
	ERG	EP111	1:50	Cell Marque
	MUC4	AG7	1:100	Cell Marque
	INI-1	MRQ27	1:50	Cell Marque

IHC expression of STAT6 was graded based on the sum of intensity (mild (1+), moderate (2+), and strong (3+)) and percentage of tumor cells showing positive immunostaining, ranging from no staining (0), < 1% (1), 1-10% (2); 11-33% (3); 34-66% (4) and ≥ 67% (5) tumor nuclei (similar to Allred scoring for Estrogen Receptor (ER)/Progesterone Receptor (PR)). Allred scoring encompasses the proportion of stained tumor cells (scored 0-5) and the intensity (score 0-3), expressed as the sum score of these two parameters.

BCOR and p53 immunostaining of the tumor cells were graded by multiplying the intensity (negative (0), weak (1), moderate (2), and strong (3)) and the extent (0% positive cells (0), rare to 33% (1), 34-66% (2), and 67-100% (3)). BCOR expression was considered high when the score was > 5, while p53 was considered high when the score was ≥ 2 [6].

Using statistical methods, the key parameters included in the risk stratification criteria were examined for any significant correlations between BCOR and/or p53 overexpression. To determine the p-value, Fischer’s Exact test was run.

## Results

The authors identified 16 patients with solitary fibrous tumors over 4 years (Table 3).

**Table 3:** Clinicopathological and immunomorphological features of solitary fibrous tumors

Criteria	Parameter	No.	%
Age	<18	0	
	≥18	16	100%
Gender	Male	9	56.25%
	Female	7	43.75%
Site	Lung/pleura	4	25%
	Non lung/pleura	11	68.75%
	Gastrosplenic & nasal cavity	1	6.25%
	Leg	1	6.25%
	Brain	2	12.5%
	Face	1	6.25%
	Kidney	1	6.25%
	Neck	1	6.25%
	Penile	1	6.25%
	Buccal mucosa	1	6.25%
	Liver	1	6.25%
	Chest wall	2	12.5%
Disease extent	Localized	11	68.75%
	Locoregional spread	2	12.5%
	Distant spread	3	18.75%
	Lymph node metastasis	2	12.5%
Tumor size	≤ 10 cm	9	56.25%
	>10 cm	7	43.75%
Mitoses	<4/10 high-power field (HPF)	12	75%
	≥4/10 HPF	4	25%
Treatment	Surgery only	7	43.75%
	Surgery with adjuvant chemotherapy	1	6.25%
	Surgery with adjuvant radiotherapy	1	6.25%
	Only chemotherapy	1	6.25%
	Only radiotherapy	-	
	Treatment incomplete	5	31.25%
Follow up (n=12)	Alive with disease (AWD)	3	18.75%
	Died of disease (DOD)	1	6.25%
	No evidence of disease (NED)	7	43.75%
	Died of other causes	1	6.25%

These cases occurred in nine men and seven women (M: F= 1.28:1), varying in age from 30-67 years (average = 52, median = 52). Among the 16 patients, tumors were located in various body sites, including pulmonary as well as extrapulmonary locations. Pulmonary SFTs (lung, mediastinum, pleura) accounted for 25% (n=4) of the cases. The extrapulmonary sites included abdomen (n=2, 12.5%), head and neck region (n=2, 12.5%), chest wall (n=2, 12.5%), lower extremities (n=1, 6.25%), brain (n=2, 12.5%), kidney (n=1, 6.25%), and penile (n=1, 6.25%).

Patients with pulmonary tumors presented with dyspnea, cough, or chest pain. The extrapulmonary tumors either exhibited pressure symptoms with swelling or site-specific symptoms like nasal bleeding or hearing loss. Imaging studies revealed a relatively well-defined heterogeneously enhancing mass with T2 hyperintensity in most cases. In Positron Emission Tomography (PET) scans effectuated in seven cases, SUVmax ranged from no active metabolism to 8.09 in the benign lesions, while the malignant SFT demonstrated an SUVmax of 12.9. There were 11 cases with localised disease, two manifested locoregional spread, one multicentric disease, and two with metastatic disease, both of which suffered lymph node metastasis. Distant metastasis included liver, adrenal, lung, and skeletal metastasis.

**Figure 1:**
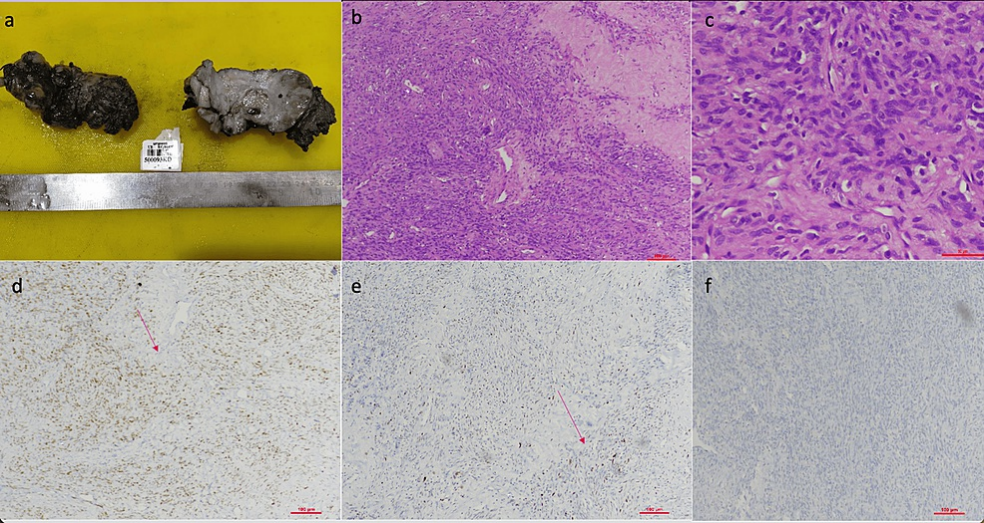
Penile mass a. Penile lesion, encapsulated and bossellated mass measuring 10x4.5x4 cm. b. hematoxylin and eosin (H&E) stain (100x) showing hypo and hypercellular areas with few staghorn-shaped vessels c. H&E stain (400x) demonstrates ovoid to spindle cells with homogenous-looking nuclei d. STAT6 immunohistochemistry (IHC) (100x) shows diffuse nuclear positive staining e. p53 IHC (100x) shows strong nuclear positive staining in nearly 70% of tumor cells f. BCOR IHC (100x) is negative.

**Figure 2:**
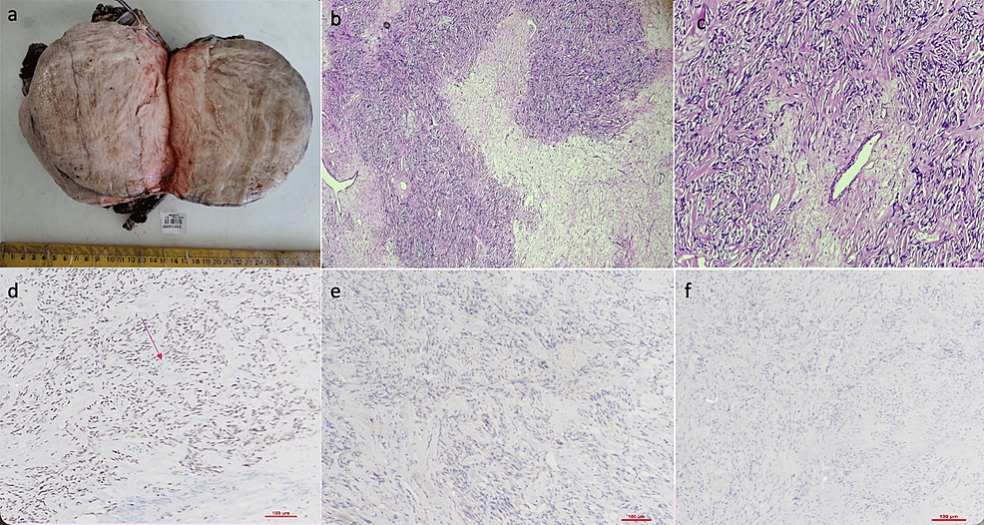
Solitary fibrous tumor of the kidney a. Gross image of kidney mass, homogenous in appearance, soft to firm in consistency b. hematoxylin and eosin (H&E) (40x) showing hypo and hypercellular areas. Native renal parenchyma is not seen. c. H&E (100x) shows haphazardly arranged spindle cells with bland nuclei d. STAT6 immunohistochemistry (IHC) (100x) shows diffuse nuclear positive staining e. BCOR IHC (100x), and f. p53 IHC (100x) are respectively negative.

**Figure 3:**
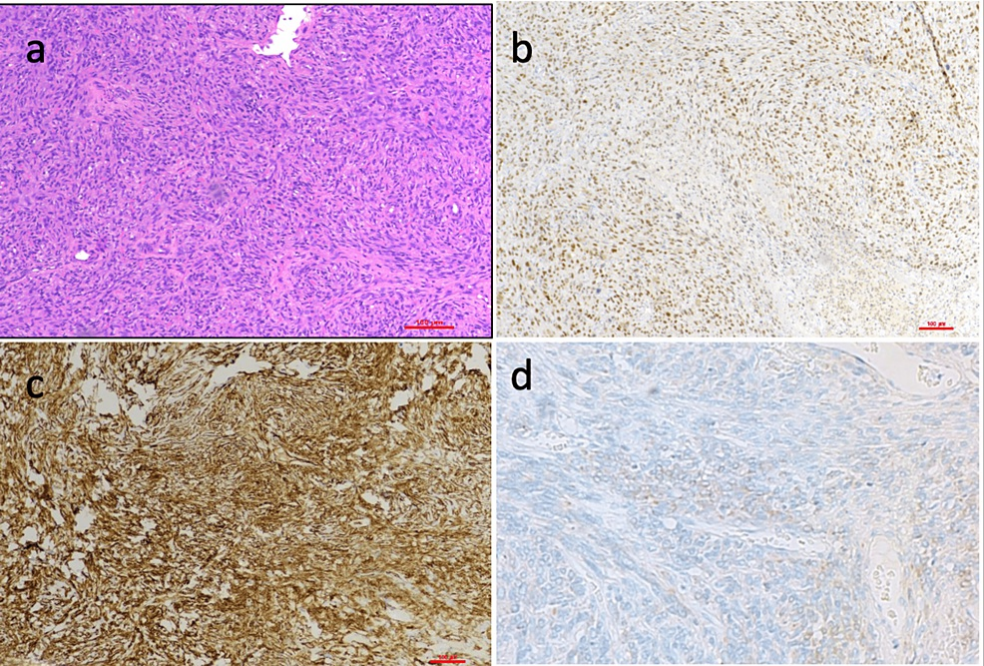
A case of solitary fibrous tumor of the lung a. Hematoxylin and eosin (H&E) (100x) showing spindle cells arranged haphazardly with a single hemangiopericytomatous (HPC)-like vessel, b. STAT6 immunohistochemistry (IHC)(100x) showing diffuse strong nuclear positive staining, c. Tumor cells are positive for CD34 IHC (100x) and d. BCL2 IHC (100x)

On gross examination of the resection specimens (n = 9), the tumors were usually well circumscribed and firm grey-white on the cut surface. Areas of haemorrhage and necrosis were usually not identified, while one case exhibited focal myxoid areas. Microscopic examination revealed 15 cases of classic SFT (Figures 1-3).

There was one case that possessed all the criteria of malignant SFT, viz., nuclear atypia, hypercellularity, necrosis, and mitoses ≥ 4/10 high-power fields (HPF) (Table 4).

**Table 4:** Criteria for malignant solitary fibrous tumors

Criterion for Malignant Solitary Fibrous Tumors
Nuclear atypia
Hypercellularity
Necrosis
Mitosis >= 4/10 high-power fields

The histopathology revealed tumor cells disposed of in fascicles and sheets with focal cellular and hypocellular areas often admixed with numerous vascular channels imparting a hemangiopericytomatous (HPC) pattern. The storiform pattern was noted in some tumors. The tumor cells expressed mild nuclear atypia with plump spindle-shaped nuclei, dispersed chromatin, inconspicuous nucleoli, and moderate cytoplasm, and ranged in size from 3.7-15.5 cm. A mitotic rate of < 4/10 HPF was observed in 12 out of 16 cases. Atypia was seen in two cases. Necrosis (>10%) was observed in one case. All the cases were stratified according to the modified Demicco and Salas risk stratification models (Table 5).

**Table 5:** Risk stratification of the cases according to modified Demicco and Salas models

	No. of cases	Percentage
Modified Demicco risk stratification		
Low risk	10	62.5%
Intermediate	5	31.25%
High	1	6.25%
Salas risk stratification models for metastases (Salas (MET))		
Very low	10	62.5%
Low	5	31.25%
Intermediate	1	6.25%
High	0	-

**Figure 4:**
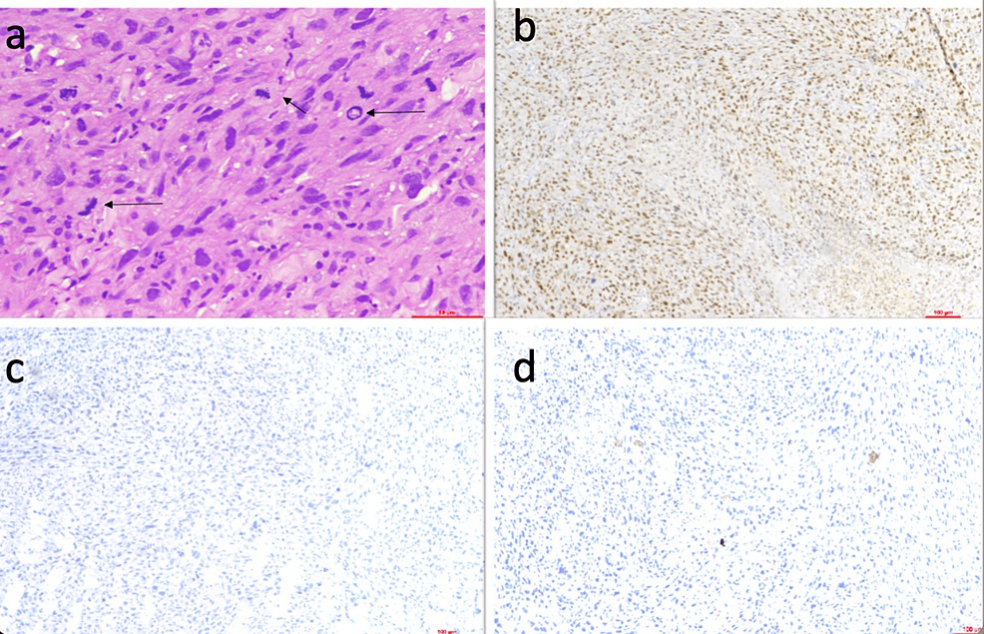
A case of malignant solitary fibrous tumor of the distal extremity a. Hematoxylin and eosin (H&E) (400x), Highly pleomorphic nuclei, with atypical mitotic figures highlighted by arrows b. STAT6 immunohistochemistry (IHC) (100x) diffuse nuclear positive c. BCOR IHC (100x) was negative (score 0) d. p53 IHC (100x) was negative (score 0).

As per the Demicco model, there were 10 cases with low risk, five with intermediate risk, and 1 high risk. As per Salas’s criteria for risk of metastasis, one case harboured intermediate risk, five cases low risk, and 10 cases very low risk of metastasis. Tumors demonstrating histological parameters of nuclear atypia, hypercellularity, necrosis and mitoses ≥ 4/10 HPF, and infiltrative growth have traditionally been termed malignant SFT [3]. One case in our series demonstrated the aforementioned histological parameters and was a 58-year-old female with distal leg swelling. On histopathology, tumor size was 10 cm in maximum dimension; the lesion was hypercellular with nuclear pleomorphism, mitoses >50/10 HPF, and focal necrosis (Figure 4); the patient also presented with metastasis in bone marrow, inguinal lymph nodes, liver, adrenal gland, lung, and D3 vertebra. As per the risk stratification of modified Demicco and Salas, this case of malignant SFT falls into the high-risk and intermediate-risk categories, respectively.

By immunohistochemistry (Table 6), STAT6 was the defining marker for the diagnosis, being positive in the tumor cells in all the cases. The tumor cells displayed a score of at least 6/8 with 13 cases (81.25%) exhibiting an immunoreactivity score of 8/8. CD34 was also positive in all the cases. CD99, FLI1, TFE3, SMA, and ERG were focally positive in a few cases, albeit of faint to moderate intensity. The prominent negative immunomarkers were AE1/AE3, desmin, beta-catenin, S100, CD31, HMB45 and TLE1. Ki67 proliferation index performed in four cases demonstrated a range of 5-15%, while the malignant SFT displayed 55-60%.

**Table 6:** Results of immunohistochemistry analysis

Immunohistochemistry		No.	%
STAT6		16	100%
CD34		16	100%
BCOR	high (score >5)	2	12.5%
BCOR	low/moderate (score ≤5)	14	87.5%
p53	high (score ≥2)	6	37.5%
p53	low (score <2)	10	62.5%

We observed BCOR and p53 overexpression in two and six cases, respectively. The two cases of overexpressed BCOR occurred in patients >55 years of age, displayed mitoses <4/10 HPF, and no necrosis, with equal distribution in the sexes and occurring respectively in the neck and chest wall. No significant correlation was witnessed between these two IHC markers and the parameters employed in the risk stratification models of modified Demicco and Salas (Tables 7, 8).

**Table 7:** Correlation of BCOR expression with clinicopathological features HPF: high-power fields

Parameter		High (n=2)	Low/moderate (n=14)
Sex	Male	1	8
	Female	1	6
Age (in years)	<55	0	7
	≥55	2	7
Size (in cms)	≤10	2	7
	>10	0	8
Location	Pleuro-pulmonary	1	5
	Abdominal/Visceral (including renal)	0	3
	Brain	0	2
	Extremity	0	1
	Head and Neck	1	2
	Penile	0	1
Mitosis (/10 HPF)	<4	2	10
	≥4	0	4
Necrosis	Present	0	1
	Absent	2	13

**Table 8:** Correlation of p53 expression with clinicopathological features HPF: high-power fields

Parameter		High (n=6)	Low (n=10)
Sex	Male	4	5
	Female	2	5
Age (in years)	<55	2	5
	≥55	4	5
Size (in cms)	≤10	5	4
	>10	1	6
Location	Pleuro-pulmonary	3	3
	Abdominal/Visceral (including renal)	0	3
	Brain	1	1
	Extremity	0	1
	Head and Neck	1	2
	Penile	1	0
Mitosis (/10 HPF)	<4	4	8
	≥4	2	2
Necrosis	Present	0	1
	Absent	6	9

The mainstay of treatment was surgery. No treatment could be offered to six patients due to a lack of financial resources or other reasons, including the malignant SFT. Resection of the tumor was executed with curative intent in nine patients, out of which one patient received adjuvant chemotherapy while another received adjuvant radiotherapy. One patient was administered chemotherapy only as the patient was unlikely to benefit from surgical excision. Due to an inoperable tumor in the temporal region, another patient could only be provided palliative radiotherapy followed by oral metronomic chemotherapy. 

On a reasonable follow-up over 2-36 months (median: 14.5 months) in 12 patients, seven (58.3%) patients manifested no evidence of disease, three (25%) patients were alive with disease, one (8.3%) patient died of other causes while a single (8.3%) patient died of disease.

## Discussion

Solitary fibrous tumors presumed to be of fibroblastic differentiation, represent a spectrum of mesenchymal tumors, encompassing tumors previously termed hemangiopericytoma, and are classified as having intermediate biological potential (rarely metastasizing) in the 2020 World Health Organization classification scheme. Patients of all ages can develop SFTs, with the highest incidence transpiring in the fifth and sixth decades of life [7]. In our study detailing 16 SFTs, the age of the patients ranged from 30-67 years (mean = 52, median = 52), which was similar to that found in the study conducted by Salguero et al [6]. These instances revealed a preference for 56.25% of males and 43.75% of females, which is close to what Demicco et al. had observed (52.8 and 47.2%, respectively) [5].

Among the 16 patients, tumors were located in various body sites, including pulmonary as well as extrapulmonary locations. SFTs of the chest (SFTC)/intrathoracic SFTs mainly consisted of SFTs of the pleura (SFTP), the lung (SFTL), and the mediastinum (SFTM), which are a subtype of SFTs [8]. SFTC were predominant (lung and pleura) and accounted for 25% (n= 4) of the cases. Demicco et al. and Salguero et al. chronicled 20.91% and 25.92% of SFTC respectively [6,5], whereas DeVito et al divulged lung/pleura as the primary tumor site in 28 (34%) cases [9].

SFTs typically exhibit no symptoms and have a slow growth rate, often detected incidentally during imaging examinations [2]. In our study, however, the patients with pulmonary tumors presented with dyspnea, cough, or chest pain. Zhang et al observed that symptoms such as pain, coughing, and difficulty in breathing often occur in the middle and late stages [8]. The extrapulmonary tumors either exhibited pressure symptoms with mass effect or site-specific symptoms like nasal bleeding or hearing loss.

Radiologically, tumors were considerably vascular, displaying an avid contrast enhancement in 65% of cases in Computed Tomography (CT) scans and, interestingly, in 35% of cases large collateral feeding vessels were verified [10]. Imaging findings in our study revealed a relatively well-defined heterogeneously enhancing mass with T2 hyperintensity in most cases. The 18F-Flurodeoxyglucose (FDG) PET scan has not been proclaimed to be a determinant in distinguishing indolent SFT (the old-fashioned typical SFT) from aggressive SFT (the old-fashioned malignant SFT) in a series of 17 patients with confirmed SFT diagnosis [11]. PET-CT available in seven cases displayed variable findings ranging from no active metabolism to hypermetabolic lesions, even in the benign SFTs. The max SUV uptake in the benign lesions recorded a highest of 8.09, while the malignant SFT unveiled a significantly increased uptake of max SUV 12.9. Thus, there is a probability that malignant behavior in SFTs may be predicted by PET-CT findings. However, more convincing evidence is necessitated to establish this facet since it could be illustrated in only a single case in our study.

Grossly, conventional SFTs are often well-circumscribed and partially encapsulated with a multinodular, whitish, firm-cut surface. Myxoid change and hemorrhage may be seen in some cases [3]. Comparable findings were registered in our study wherever resection samples were available. Necrosis though absent in typical SFTs can be encountered in malignant SFTs.

On histopathology, SFTs are variably cellular tumors formed of ovoid to spindled cells with a patternless or a storiform pattern against a variably collagenous stroma having thin-walled large branching “staghorn”-shaped (HPC-like) blood vessels. Perivascular fibrosis is also frequently evident in medium-sized blood arteries. The stroma in the background may exhibit localised or widespread myxoid alteration [4,12,13,14]. Classical fibrous SFTs are paucicellular tumors composed of spindle-shaped cells that are grouped in short, wavy fascicles or dispersed randomly within a conspicuous fibrous stroma that exhibits cracking artefacts and an abundance of keloid type of collagen. The nuclei of these cells are homogeneous, elongated, or fusiform, and the cytoplasm is sparse [15,16].

The endothelial cells in the vascular channels merge with the surrounding tumor cells since there is no connective tissue layer. Perivascular fibrosis or background fibrosis is not usually encountered. Typically mitotic activity is infrequent and nuclear pleomorphism and necrosis are absent or minimal. Cellular SFTs are more cellular, with spindle-shaped and rounded cells, round to oval nuclei, and condensed chromatin. Perivascular fibrosis is common while gaping blood vessels are more frequent around the perimeter of tumors. A markedly cellular tumor comprises sheets of round, more primitive-looking cells. HPC-like vasculature is not pathognomonic for SFT and can be nonchalantly discernible in other mesenchymal tumors, including numerous soft tissue sarcomas [17]. Meningeal SFTs conspicuously exhibit HPC-type morphology and are more cellular than pleural and extra-pleural tumors [4,18].

Some SFT variations such as fat-forming, giant cell-rich, and dedifferentiated types evince several peculiar morphological characteristics. Mature adipocytes are abundant in the stroma of the fat-forming type of SFT, often affecting deep soft tissues, but have also been observed in the paratesticular soft tissue, neck, mediastinum, and stomach [19-23]. The giant-cell type of SFT flaunts numerous multinucleated stromal giant cells, typically clustered around the pseudovascular spaces. Although most of these cases involve soft tissue in the periorbital region, a few have also been documented in extraorbital sites, including the head and neck, retroperitoneum, back, vulva, hip, and inguinal region [24-26].

The dedifferentiated variety, which is incredibly uncommon, portrays a sudden transition to a low- or high-grade sarcoma with neighbouring conventional SFT. The dedifferentiated component typically manifests as undifferentiated pleomorphic sarcoma or spindle cell sarcoma not otherwise characterized, with rare instances of osteosarcomatous or rhabdomyosarcomatous differentiation. Recurrent as well as primary tumors can express this dedifferentiation and may be attributed to more recent molecular changes and are associated with loss of immunohistochemical marker expression. There have also been reports of neuroendocrine and squamous differentiation in SFTs [27-31].

The tumors ranged in size from 3.7 to 15.5 cm in our study. Mitotic rate < 4/10 HPF was observed in 75% (n = 12) of cases. Atypia was identified in 12.5% (n = 2) of cases and necrosis in 6.25% (n =1) of cases. Similar findings were documented in various other studies across the literature [5,6,32]. In the study conducted by Machado et al, 38.1% of tumors revealed hypercellularity with a predominant patternless and/or HPC-like growth pattern, 13.4% disclosed ≥4 mitoses/10 HPF, and 16.5% evinced necrosis [13].

Owing to the diverse histological features and the involvement of varied anatomic locations, SFTs can mimic other soft tissue neoplasms of different lineages. Various diagnostic differentials of typical SFT considered in our study, especially on core biopsies included commonly synovial sarcoma, gastrointestinal stromal tumor (GIST) and dermatofibrosarcoma protuberans (DFSP), and also occasionally schwannoma, cellular hemangioma, fibromatosis, myoepithelioma and leiomyoma, more so at uncommon sites for SFTs. Malignant peripheral nerve sheath tumor (MPNST), melanoma, synovial sarcoma and at times DFSP also need to be primarily excluded before arriving at the diagnosis of traditionally described malignant SFT (Table 9) [33-37].

**Table 9:** Differential diagnoses (DDs) considered in our study with regard to the cytoarchitecture

DDs of solitary fibrous tumors	Site	Histopathology	Immunohistochemistry	Genetic alterations
Synovial sarcoma (SS)	Most often deep soft tissues of extremities or limb girdles, distal extremities (fingers, hand, foot), Head and & Neck	Sheet-like growth pattern, spindle-to-ovoid cell morphology, HPC-like vasculature in certain cases	TLE1+, CD99+, BcL2+ EMA+, CK+, STAT6-	SYT-SSX1/2 rearrangement
Malignant peripheral nerve sheath tumor (MPNST)	Trunk and extremities, followed by the head and neck area	Cellular sheets of spindle cells, which can be interspersed with areas of low cellularity. characteristic morphology resembling nerve sheath structures, at least in certain focal areas. The tumor cells may exhibit HPC-like vasculature and appear more prominent around blood vessels.	S100 + (<50%), SOX10 + (<70%) GFAP+ (20–30%) H3K27me3 Loss, STAT6-	NF1, CDKN2A/ CDKN2B, and PRC2 core components (EED or SUZ12) mutations
Gastrointestinal stromal tumor (GIST)	Gastrointestinal tract, omentum, mesentery, retroperitoneum or pleura	Spindle to ovoid cells organized in bundles or scattered throughout.	DOG1+, C-KIT+, STAT6-	KIT and PDGFRA mutations, SDH deficient
Melanoma	skin, mucosa, internal organs	Epithelioid / spindle shaped cell with nuclear pleomorphism and enlargement and prominent nucleoli	S100+, SOX10+, MelanA+, MART1+, HMB45+, STAT6-	BRAF V600E mutation
Dermatofibrosarcoma protuberans (DFSP)	Trunk, proximal extremities, head and neck region, genital area, the breast, and at acral sites	Dermal uniform spindle cells arranged in storiform pattern, whorls & short fascicles.	CD34+, STAT6 -	COL1A-PDGFB fusion
Schwannoma	Paravertebral, retroperitoneum, pelvis & mediastinum	Predominantly or exclusively composed of Antoni A areas with interlacing fascicles of spindle cells having tapered nuclei. Hyalinized vessels are focally seen.	S100+, SOX10+, STAT6-	NF2 mutations
Cellular hemangioma	face, scalp, chest or back, viscera	Lobules of capillary sized vascular channels, lined by single layer of endothelial cells	CD34+, CD31+, ERG+, STAT6-	
Fibromatosis	Deep soft tissue	Long, sweeping fascicles with thin-walled vessels; bland cells with mild to moderate cellularity and minimal atypia. May show staghorn vessels.	Beta catenin+, SMA+, STAT6-, CD34-	CTNNB1 and APC gene mutations
Myoepithelioma	Salivary gland, breast, soft tissue	Spindled, epithelioid, plasmacytoid, clear or oncocytic cells with hyalinized, fibrous, myxoid, or mucoid stroma	GFAP+, S100+, SMA+, calponin+, p63+, STAT6-, CD34-	EWSR1 rearrangement, NTF3-PLAG1, FBXO32-PLAG1 and GEM-PLAG1 fusions.
Leiomyoma	Deep soft tissue, subcutis	Bundles or fascicles of spindled cells with eosinophilic and possibly fibrillary cytoplasm with blunt-ended nuclei, indistinct nucleolus and variable cytoplasmic vacuoles at one end	SMA+, h-caldesmon+, CD34-, STAT6-	

Other neoplasms such as spindle cell lipoma, well- and dedifferentiated liposarcoma (WDL/DDL), and angiofibroma to name a few can also complicate the diagnostic quandaries concerning SFTs, as discussed in Table 10. In fact, given the aforementioned diagnostic pitfalls and the ambiguous nature of the histopathological characteristics of SFTs, Jain et al advance a step further to emphasize the appraisal of SFTs by experienced soft tissue pathologists [38]. Nonetheless, a pertinent point the authors wish to underline here is that at unusual sites for SFTs, the more familiar entities at those locations should be verily excluded before proffering a diagnosis of SFT.

**Table 10:** Other neoplasms that can also cause diagnostic dilemmas with solitary fibrous tumors

Differential Diagnoses	Site	Histopathology	Immunohistochemistry	Genetic alterations
Well- and dedifferentiated liposarcoma (WDL/DDL)	Retroperitoneum, spermatic cord, and (more rarely) mediastinum, head and neck, and trunk	Fascicles or sheets of atypical spindle cells along with lipogenic component	MDM2+, CDK4+, p16+, CD34+/−, STAT6−/+	MDM2 & CDK4 amplification
Spindle cell lipoma	Subcutis of posterior neck, upper back & shoulder, face, scalp, orbit, oral cavity & rarely extremities	Short fascicles of bland spindle cells with short stubby nuclei, variable number of adipocytes, and ropy collagen bundles; fibromyxoid stroma, mast cells	CD34+, STAT6-	RB1 deletion
Malignant mesothelioma	Pleura, Peritoneum	Fascicles or haphazardly distributed atypical spindle cells with increased mitoses; densely collagenized stroma with hypocellular atypical spindle cells in desmoplastic mesothelioma	CK+, EMA+, D2-40 +, calretinin+, WT1 +, STAT6-	BAP mutation
Sarcomatoid carcinoma	Pleura, Peritoneum	Predominantly anaplastic spindle cells	CK+, STAT6-	TP53 and KMT2D mutations
Angiofibroma	Usually subcutis of extremities, particularly involving around large joints like knee	Variably myxoid to collagenous stroma, branching capillary network & uniform bland spindle cells with ovoid or tapering nuclei; perivascular collagenization	EMA −/+, CD34 −/+ STAT6-	NCOA2 gene rearrangements
Myofibromas	Skin & subcutis of extremities, head & neck & trunk. Infantile cases involve liver, heart, gastrointestinal tract (GIT), brain & bone	Distinctive biphasic pattern with nodules comprising immature spindle cells in the center with HPC-like vasculature and whorls of myoid cells at the periphery with a basophilic pseudo-chondroid appearance	SMA+/−, CD34 −/+, desmin +	PDGFRB alterations
Deep fibrous histiocytoma	Extremities followed by head and neck region	Uniformly cellular, storiform to short fascicular pattern of plump spindle cells	CD34 + (40%), SMA +/−, STAT6-	---
Meningioma, primarily fibroblastic variant	Meninges, spine, lung, upper extremities, mediastinum	Spindle cell morphology and fascicular pattern in a collagenous stroma. Atypical and anaplastic meningioma can mimic malignant SFT due to the presence of patternless/ sheet-like growth, necrosis, frequent mitoses, nucleolar prominence, nuclear pleomorphism, and small cell change.	SSTR2a+, PR+, EMA+, CD34-, STAT6-	NF2 mutation

All the cases in the current study were immunoreactive for STAT6 and CD34, a finding catalogued in most of the studies in the literature. STAT6 has been identified as a rewarding IHC antibody marker for the diagnosis of SFT, with its sensitivity ranging from 98-100% [39]. Immunohistochemical tests for STAT6 utilizing antibodies against the C-terminus in SFTs highlight the NAB2-STAT6 fusion protein, which is localized to the nucleus. However, nuclear staining for STAT6 has also been observed in other mesenchymal tumors such as unclassified sarcomas of spindle cell or epithelioid morphology, desmoid tumor, well-differentiated and de-differentiated liposarcoma, neurofibroma, synovial sarcoma, clear cell sarcoma, myxoid liposarcoma, alveolar soft part sarcoma, angiofibroma, angiosarcoma, DFSP, epithelioid sarcoma, fibrosarcoma, GIST, hemangioma, leiomyoma, leiomyosarcoma, low-grade fibromyxoid sarcoma, MPNST, myofibroma, myxoma, osteosarcoma and sinonasal glomangiopericytoma [40-41].

Fortunately, almost all the above-mentioned tumors display focal/weak nuclear staining. Thus, it is paramount not only to describe the nuclear staining of STAT6 but also to consider the proportion of tumor cells as well as the intensity of staining. The incorporation of a staining score for STAT6 in our study, similar to the Allred staining score used in breast carcinomas, seems rational to hammer home the aforementioned essence. Moderate to strong nuclear staining has been spotted in unclassified sarcomas, desmoid tumors, and WDL/DDLs. But these tumors can be segregated from SFTs based on the clinico-radiological and more importantly histopathological features. Therefore, the presence of STAT6 in SFTs is only significant when it is present in a diffuse and strong nuclear staining pattern, in correlation with the histological findings. Rarely, lipomatous SFT may mimic WDL, and in the event of STAT6 expression in such cases, WDL can be distinguished by the co-expression of MDM2 and CDK4, demonstrable by immunohistochemistry and/or molecular evidence of gene amplification. In our study, CD99, FLI1, TFE3, SMA, and ERG were focally positive in a few cases, albeit of faint to moderate intensity.

Nuclear immunoreactivity for TFE3 was found in 44-50 (88%) SFTs in a study conducted by Zhou et al [42]. Since relatively few soft tissue tumors are TFE3 positive, TFE3 is fruitful as a diagnostic marker for SFT and can be paired with STAT6 for a more accurate diagnosis. CD34 expression has been chronicled in 81-95% of SFTs but the expression is lost especially in malignant and dedifferentiated tumors. BCL-2 is a more sensitive marker (> 90% sensitivity) while CD99 is less sensitive (~75% sensitivity). However, the specificity of both these markers is quite low [41]. Geramizadeh et al proposed CD99 to be the least sensitive and CD34 to be the least specific markers for SFTs [43]. Tariq et al and Wei et alechoed similar observations with respect to the immunoreactivities of CD34, SMA, and CD99 in SFTs [41,44]. Miettinen et al found ERG to be consistently negative in SFTs [45], while Lee et al reported ERG positivity in a dedifferentiated SFT of the parotid gland [46].

Argani et al discovered strong BCOR nuclear expression by IHC in malignant SFTs of the kidney with undifferentiated/small round cell phenotype accompanied by the characteristic branching vasculature [47]. Salguero-Aranda et al evaluated BCOR expression, wherein 40% of SFTs evinced a high expression [6]; however, BCOR expression did not reveal any prognostic value, although it may be beneficial as an additional IHC marker for SFT. Taking a cue from this study, we evaluated BCOR expression in all 16 cases of SFT, out of which 12.5% (n=2) demonstrated overexpression. These two cases were neither associated with features of malignancy/atypical morphology nor did they arise from the kidney. Therefore, in our investigation, there was no discernible relationship between BCOR overexpression and any important SFT parameter.

P53 mutation was detected in 17% of the study conducted by Machado et al [13]. In the series of Salguero-Aranda et al, p53 was high in 50% (22 out of 44) of the cases [6]. The correlation between p53 IHC expression and mitotic count, an indicator of malignancy as explained in the study by Park et al [48], was indeed observed. However, Salguero-Aranda et al could not establish any connection of p53 with other pathological parameters, a finding dissimilar to that discovered by Schirosi et al [49]. With the same method of interpretation of p53, the authors found p53 high in 6 (37.5%) cases. However, no significant correlation was detected in our study between p53 overexpression and any significant atypical histological parameter of SFT or those considered in the risk stratification models.

Resection of the tumor was done with curative intent in 56.25% of cases in our study, whereas in the analysis shepherded by Kim et al, 80.43% of cases underwent resection. Complete resection is a salient factor affecting local recurrence and metastasis [18]. In addition to surgery, radiofrequency ablation and photodynamic therapy can be employed for unresectable tumors. There is no universal agreement on how to treat SFTs due to their rarity and absence of randomized control trials. For the treatment and management of these tumors, a multidisciplinary team approach is advised [43].

On a median follow-up of 90 months, 9.3% recurred, 11.3% metastasized, 10.3% died of disease and 76.2% were free of disease in the study undertaken by Machado et al [13]. According to Rekhi et al, over a period of 3-104 months (median = 21), clinical follow-up was available for 20/33 patients (60.6%) with six instances of tumor recurrences and one metastasis; 11 patients were deemed to be alive with no evidence of disease (AWNED), three patients alive with disease (AWD) and six patients died of disease (DOD) [50].

One study registered a higher occurrence of local recurrence and distant metastasis in malignant SFTs [51]. Conversely, another study uncovered that tumors lacking malignant histological features exhibited aggressive behavior, while those with malignant histological features displayed an indolent nature [52-53]. The location of the tumor outside the thoracic region independently predicted a poorer prognosis. Tumors situated in the meninges, pelvis, retroperitoneum, and mediastinum were associated with an increased risk of recurrence [49]. Among 39 cases examined in a particular study, local recurrence was observed in 13 cases (39.3%) [17]. Distant metastasis served as a prognostic indicator, as 75% of patients with metastatic SFTs succumbed to the disease [54]. In a study focusing on extra-thoracic SFTs, the median overall survival duration ranged from 59-94 months, with 5-year and 10-year survival rates of 89% and 73%, respectively [55].

 Among the cohort of Georgiesh et al, 96 patients (33%) experienced disease recurrence, with a median time to recurrence of 36 months (ranging from 2-210 months). Notably, 31% of recurrences manifested at least 5 years after the initial surgery. Distant metastasis was noted in 70 patients (24%), and the median time to distant recurrence was 43 months (ranging from 2-298 months). Lung was the most common initial site of metastasis, followed by multiple sites such as liver, and bone [56].

Considering that the majority of SFT cases do not result in patient mortality, Reisenauer et al opined that progression-free survival (PFS) might be a more practical endpoint to consider instead of overall survival (OS) [32]. However, due to the low recurrence rate, it is challenging to gather a study population with sufficient size to yield meaningful statistical data. Moreover, in a study like theirs, even with a median follow-up of over 5 years, the period of surveillance might not capture all instances of recurrence, as the time to recurrence can exceed the duration of observation. Baldi et al. discovered a median time to recurrence of 12 years following the primary lesion, emphasizing the importance of long-term follow-up for all patients diagnosed with SFT [57].

In the research orchestrated by Salas et al, 20 (12.3%) patients experienced local recurrence, and 27 (16.7%) patients suffered metastatic recurrence on a median follow-up period of 32.8 months. Furthermore, 50% of local recurrence and metastases occurred at 4.3 and 3.6 years, respectively, after initial resection [58]. This study unequivocally demonstrates that various prognostic SFT subgroups may benefit from assorted therapy approaches, and the key issue at hand is how these groups should be handled. Against this backdrop, the significance of the proposed risk stratification models was accentuated by Salas et al [58] and Demicco et al [55].

All the cases in the present study were stratified according to the modified Demicco and Salas risk stratification model (Salas (MET)). As per the Demicco model, there were 10 (62.5%) with low risk, five (31.25%) with intermediate risk, and one (6.25%) with high risk. In the research comparing diverse risk stratification systems by Demicco et al, 163 (61%) of the cases were considered to be low risk, and 31 (12%) were considered to be high risk [5]. As per the Salas (MET), the authors encountered one case (6.25%) with intermediate risk, five (31.25%) cases with low risk, and 10 (62.5%) cases with very low risk of metastasis. Based on Salas’s criteria, Demicco et al found that 2.3% of patients had a high likelihood of metastasis, 20.5% had an intermediate likelihood, 42.9% had a low likelihood, and 34% had a very low likelihood [5]. Both the Demicco model and the Salas (MET) model correlated with time to first metastasis [5]. The single case satisfying the malignant criteria for SFT fell in the high-risk category of Demicco and the intermediate-risk category of Salas. The reason for this minor discordance was the age of the patient; had the age been 60 years, this case also would have been classified as high risk according to Salas criteria. Thus, it can be opined that both the risk stratification models produced similar results when applied in our cases, and thereby can be reliably utilized for predicting malignant behavior in SFTs. A G-score model based on mitotic count, necrosis, and gender was envisaged by Georgiesh et al to be deployed as a predictor of early and late recurrence in SFT. They contend that it outperforms previous models in identifying people with a low risk of relapse and also propose a follow-up schedule based on the G-score, and for patients with a low recurrence risk by G-score, a less rigorous follow-up program might be contemplated. Patients in the intermediate risk category deserve enhanced prediction of prognosis and NAB2-STAT6 fusion variants can have a definitive role in the risk classification. [56]

Thus, it may sound judicious to develop alternate management strategies, such as considering patients with favorable prognostic factors as having a lower risk of recurrence following surgery, and thereby reserving them for exclusive monitoring with a lengthy follow-up; on the contrary, triaging patients with more aggressive tumors for radiotherapy and/or systemic treatment. This could hence evolve in the generation of a survival calculator in SFTs for adapting treatments based on individual clinical circumstances.

Notwithstanding the above-ventilated details, our study endured certain limitations, being retrospective within a single tertiary cancer center, involving a small number of cases, and over a short period.

## Conclusions

SFTs though relatively uncommon should be considered as a differential diagnosis of any spindle cell neoplasm with sheet-like haphazard growth pattern, ovoid to spindle cell morphology, and hemangiopericytoma-like vasculature even at extrapulmonary sites, while malignant SFTs can compound the diagnostic conundrums of spindle cell sarcomas. In fact, SFTs, by and large, can pose as ‘the great simulator’ of soft tissue tumors. Immunohistochemistry is vital for veracious recognition, especially STAT6, and a scoring system for this marker can be fashioned for better delineation. SFTs can behave erratically clinically and thus, the risk assessment stratification expressed in our study based on the models developed by Salas and/or Demicco can be efficacious for classifying patients for clinical trials. These concepts can serve as a foundation for future studies incorporating biomarkers such as NAB2-STAT6 fusion transcript types into the risk assessment model and also for the establishment of targeted therapy (since the tumor harbors a particular fusion product), especially in malignant cases. Although many oncological diseases are tracked for a limited period, such as 5 years, SFTs have demonstrated recurrence much later in life, several years after initial diagnosis and treatment, emphasizing the need for lifelong follow-up.
